# Television Watching, Diet Quality, and Physical Activity and Diabetes among Three Ethnicities in the United States

**DOI:** 10.1155/2012/191465

**Published:** 2012-07-17

**Authors:** Fatma G. Huffman, Joan A. Vaccaro, Joel C. Exebio, Gustavo G. Zarini, Timothy Katz, Zisca Dixon

**Affiliations:** Department of Dietetics and Nutrition, Robert Stempel College of Public Health and Social Work, Florida International University, AHC1-435, Miami, FL 33199, USA

## Abstract

Diabetes is a world-wide epidemic associated with multiple environmental factors. Prolonged television viewing (TV) time has been related to increased risk of obesity and type 2 diabetes in several studies. TV viewing has been positively associated with cardiovascular disease risk factors, lower energy expenditure, over-eating high-calorie and high-fat foods. The objective of this study was to assess the associations of hours of TV viewing with dietary quality, obesity and physical activity for three ethnic minorities with and without type 2 diabetes. Diet quality and physical activity were inversely related to prolonged TV viewing. African Americans and participants with type 2 diabetes were more likely to watch more than 4 hours of TV per day as compared to their counterparts. Diet quality was inversely associated with physical activity level. Future studies are needed to establish the risk factors of prolonged TV watching in adult populations for the development of diabetes or diabetes-related complications. Although strategies to reduce TV watching have been proven effective among children, few trials have been conducted in adults. Intervention trials aimed at reducing TV viewing targeting people with type 2 diabetes may be beneficial to improve dietary quality and physical activity, which may reduce diabetes complications.

## 1. Introduction

Diabetes has become a worldwide epidemic. Quality of diet and physical inactivity have been attributed to the rise in prevalence of obesity leading to type 2 diabetes (the most common form) among developed and developing countries. Prolonged television (TV) viewing time has been associated with increased risk of obesity and type 2 diabetes in men from the Health Professionals Follow-Up Study (HPFS) [1] and women from the Nurses' Health Study [2]. Average hours of TV viewing, independent of physical activity, was positively associated with low-density lipoprotein cholesterol and inversely associated with high-density lipoprotein cholesterol and apolipoprotein A1 in an eight-year prospective study of 486 men randomly sampled from the HPFS [3]. Several other studies reported TV watching as positively associated with cardiovascular risk factors such as blood pressure, low-density lipoprotein, triglycerides, and insulin resistance [4–6] and overeating [7]. The average hours of TV viewing was estimated at 2.73 hours per day for all U.S. civilians and 3.44 hours for those who reported watching TV according to the Bureau of Labor Statistics [8]. Estimates for adults are close to 5 hours per day [9]. 

The association of TV with poor health outcomes may be partially attributed to the role of television. Although sedentary behaviors such as computer use, reading, board and video game-playing, and driving a car require little physical exertion, TV viewing results in a lower metabolic rate than other sedentary activities [10]. Unique to TV, as compared to other sedentary activities, is food commercials. Lower intakes of fruits, vegetables, and whole grains and higher intake of fried foods, processed meat and sugar-sweetened beverages have been associated with TV viewing in adults [11]; and references therein. Exposure to food advertisements has been associated with overeating and choosing foods of poor dietary quality (higher salt, saturated fat, and lower dietary fiber) [1, 2]. 

Analysis of diet by a dietary index provides a more comprehensive approach to food consumption and health than measurement of single nutrients or diet quantity [12, 13]. Dietary indexes have been developed to measure the degree of adherence to a standard deemed as “healthy eating.” A dietary index includes the most important dietary components of a particular diet and then scores adherence to those components. The dietary index chosen for this study, the Healthy Eating Index, 2005 (HEI-05) (discussed in detail in Section 3.2), parallels the dietary recommendations for persons at risk for or with diabetes. 

Common types of physical activities consist of leisure-time (including sports and social activities), occupational, transport, and at-home or domestic physical work. While physical inactivity is an established risk factor for obesity and diabetes, TV viewing hours has not been established as a risk factor for obesity and diabetes in adult populations. Despite the evidence by prospective studies that high levels of TV viewing was a risk factor for improper dietary intake, poor lipid profile, and type 2 diabetes in both men and women, public health goals target increasing physical activity, but has not set thresholds for sedentary activities [14]. Little or no correlation has been found for average hours of TV viewing and hours of physical activity for large epidemiological studies in men [3] and women [2]; yet, obesity indicators were positively associated with TV hours in these studies. Evidence suggests that TV watching may be an independent risk factor for obesity and type 2 diabetes. 

## 2. Purpose

The objective of this study was to assess the associations of hours of TV viewing with dietary quality, obesity and physical activity for three ethnic minorities with and without type 2 diabetes. Since median TV viewing was 3 hours and the 75th percentile was 4 hours for the combined sample, high TV viewing was set at more than 4 hours. Sedentary behavior is a risk factor for diabetes. Those persons with diabetes should be more likely to engage in sedentary behavior such as TV viewing. Therefore, the following hypotheses were tested. (1) Persons with type 2 diabetes will be more likely to watch more than 4 hours of TV per day as compared to persons without diabetes, independent of ethnicity; (2a) individuals with type 2 diabetes in the lowest quartile of HEI-05 (poorest diet) will be more likely to watch over 4 hours of TV as compared to those in the highest quartile, independent of ethnicity; and (2b) participants with the highest quartile of physical activity measured as metabolic equivalents (MET) per hour per week (1 kcal*·*kg^−1^ *·*h^−1^) will be less likely to watch more than 4 hours of TV as compared to those in the lowest quartile, independent of diabetes status and ethnicity. Physical activity was estimated by self-reported responses from the Modifiable Activities Questionnaire [15] (further details are provided in Section 3.2). Hypothesis 2 was tested with physical activity and diet as covariates so each variable would adjust for the other. A secondary purpose was to assess differences in dietary components of the HEI across ethnicities, physical activity level, BMI, and TV viewing by ANOVA models.

## 3. Materials and Methods

### 3.1. Study Participants

This was a cross-sectional study of *N* = 868 participants: Cuban American (CA) (*N* = 361), Haitian American (HA) (*N* = 258), and African American (AA) (*N* = 249) with (*N* = 455) and without (*N* = 410) type 2 diabetes collected from 2008–2010. By study design, approximately half of each racial/ethnic minority in this study had type 2 diabetes and the rest were free of diabetes. Sample size was determined by a pilot study of cardiovascular risk factors for CA and was based on 80% power to attain a difference with 95% confidence among 7 subgroups [three ethnicities, two genders and diabetes status (with and without diabetes)], for variations in serum glucose, hemoglobin A1C, and blood lipids. Recruitment of CA was conducted based on a target of *N* = 350. Sufficient power was achieved at *N* = 250. The remaining racial/ethnic minority target goal was lowered to *N* = 250. When the target was achieved recruitment efforts were ended. Recruitment was conducted by alternating between selecting potential participants with and then without type 2 diabetes. Participants were initially recruited by randomly generated mailing lists. The lists of addresses were purchased from Knowledge Base Marketing, Inc., Richardson, TX, USA This company provided four mailing lists generated from multiple databases of CA and AA, identified as having or not having type 2 diabetes from Miami-Dade and Broward Counties, Florida, USA During a one year period, approximately 10,000 letters, in English and Spanish, explaining the study and containing contact information with an invitation flyer, were mailed to CA individuals with and without type 2 diabetes. Three percent (*n* = 300) of the letters were returned due to unknown addresses. From the remaining delivered letters, 4%  (*n* = 388) responded. About 7,550 letters were mailed to AA participants with and without type 2 diabetes. Approximately, 6.3% (*n* = 477) of the letters were returned due to unknown addresses. From the remaining delivered letters, 4%  (*n* = 256) responded. We were not able to recruit HA in the same manner because Knowledge Base Marketing, Inc. did not have a database for the HA community. Therefore, recruitment of HA participants (*n* = 258) were from community-based sources. (a) Local diabetes educators and community health practitioners in Miami-Dade and Broward Counties were contacted: several local diabetes educators who were either former students or in close contact with the Department of Dietetics and Nutrition at Florida International University (FIU). Official letters of invitation outlining the study were mailed to the diabetes educators and health professionals in Miami-Dade and Broward counties requesting their cooperation in recruiting individuals. (b) Invitational flyers were distributed to all university faculty, staff and students using the university-wide e-mail system explaining the research protocol and requesting their assistance in the study. (c) Several residential rental facilities also agreed to help in the recruitment process. (d) Print advertisements were placed in local Haitian newspapers and principal gathering places of these groups such as churches, supermarkets, and restaurants. Radio advertisements on local Creole stations were also aired. When the target recruitment goal was reached for all ethnic groups, efforts to recruit participants were stopped. Interested participants were initially interviewed by telephone, where the study purpose was explained to them. Age, gender, ethnicity, diabetes status and study qualification were determined. To ascertain type 2 diabetes status, each participant who self reported having diabetes was asked for the age of diagnosis, and initial treatment modalities. Participants who did not qualify for the study (*n* = 28) consisted of those being younger than 35 years old  (*n* = 12), other ethnicity (*n* = 5), or having other chronic diseases or illnesses (*n* = 11). If a subject was determined to be eligible, then his or her participation was requested at the Human Nutrition Laboratory at Florida International University (FIU). Participants were instructed to refrain from smoking, consuming any food or beverages except water, and engaging in any unusual exercise for at least eight hours prior to their blood collection. This study was approved by the Institutional Review Board at FIU. The purpose and protocol of the study were explained to the participants, and their written consent either in English, Spanish, or Creole was obtained prior to the commencement of the study. Laboratory results showed that nineteen participants (CA = 7; HA = 8; AA = 4) who reported not having diabetes were reclassified as having type 2 diabetes according to American Diabetes Association standards. These participants were given their laboratory results and referred to their physicians. This study adhered to the institutional review board requirements on the use of human subjects. Trained interviewers who were bilingual in English/Creole and English/Spanish administered questionnaires. 

### 3.2. Measures

Sociodemographic data was collected by self-report with a questionnaire constructed by the principal investigator. This questionnaire included questions about age, gender, tobacco use, education, income, language preference, years lived in the United States, and health insurance. 

Physical activity and TV viewing were estimated from the Modifiable Activity Questionnaire (MAQ) [15]. The use of this questionnaire to estimate metabolic cost and perform a MET calculation is explained in detail in the compendium of physical activity [16]. The MAQ assesses leisure activities from a popular list, (such as dancing swimming, bicycling, walking/jogging (outdoor, treadmill), strength/weight training, aerobics (water, dance, step), gardening or yard work) over the past year that were performed more than 10 times. TV viewing was reported to the nearest 1/4 hour. The participants, with the help of a trained interviewer, estimate the number of months and frequency per month and duration of each activity. For occupational activity, participants reported all jobs held over the past year. The number of hours sitting at work and usual mode of transportation to work was recorded for each job. Total physical activity was calculated, summing all leisure and occupational activity hours per week. These values were then multiplied by their estimated metabolic cost. Activity was expressed as metabolic equivalents (MET) per hour per week (1 kcal*·*kg^−1^ *·*h^−1^). One MET is the equivalent of sitting quietly. 

Anthropometric measures taken included waist circumference, weight, and height. Waist circumference to the nearest 0.1 cm was measured horizontally with a nonstretchable measuring tape. The tape was placed midway between the 12th rib and iliac crest at minimal respiration to determine central obesity. Body mass index(es) (BMI) was calculated by weight divided by height (kg/m^2^). 

Glycosylated hemoglobin (A1C) was measured from a 20 ml sample of venous blood collected from each participant after an overnight fast (8–10 h) by a certified phlebotomist using standard laboratory techniques. Whole-blood samples for A1C were collected in a tube containing EDTA and A1C percentages were measured using Roche Tina Quant method by the Laboratory Corporation of America (LabCorp, Miami, FL, USA). 

Dietary intake was measured using the semiquantitative food-frequency questionnaire, version 97GP 2006, developed by Walter C. Willett (copyrighted at Harvard University, Boston, MA, USA). The FFQ has been extensively validated and standardized in multiethnic population-based prospective and cross-sectional studies [17–19]. Participants self-reported average consumption of specific amounts of various foods over the past year, choosing from a frequency range from “never” to “six or more servings” per day, based on standardized portion sizes. The participants were shown food models and asked to estimate the number of servings that they consumed for each portion. In addition, this FFQ includes questions about type and frequency of vitamin and mineral supplementation, alcohol consumption and details about sugar, salt, and fat used in cooking and as condiments. Open-ended section for ethnically specific foods was included in this version of the FFQ. The questionnaire was reviewed by the interviewer with the participant for omissions at the close of the interview. The Harvard University Food Composition Database was used as the reference for macro- and micro-nutrient calculations. Daily servings of food groups were calculated by summing frequency factors (which corresponded to the reported consumption frequencies) for all food items.

Dietary quality was assessed by the HEI-05, which measures adherence of the individual's overall diet to the Federal Dietary Guidelines of the United States Department of Agriculture (USDA). The HEI-05 was formulated based on the USDA food guide pyramid using the proportions of grains, fruits and vegetables, dairy, meat, and discretionary sweets, fats, and oils. Diet quality from the HEI-05 was calculated from each completed FFQ. The HEI-05 consists of 12 components—total fruit, whole fruit, total vegetables, dark green and orange vegetables and legumes, total grains, whole grains, milk, meat and beans, oils, saturated fat, sodium, and energy from solid fats, alcohol, and added sugars (SoFAAS). Twelve individual components of the HEI-05 represent all of the major food groups found in MyPyramid (an online, interactive counterpart based on the DGA's available to the public). The intakes of foods and nutrients are represented on a density basis, as amounts per 1,000 calories [13]. A minimum score of zero for no intake and a maximum score of either 5 for fruits, vegetables, and grains, 10 for milk and meat/bean products, and 10 for oils was assigned for healthy components. For sugars, saturated fat, and sodium, higher scores were assigned for lower intakes. For SoFAAS the highest score was 20 (representing ≤ 20% of energy) and the maximum for saturated fat and sodium was 10 points each. Saturated fat and sodium received a score of 8 for the intake levels that reflect the 2005 and 2010 DGA's less than 10% of energy from saturated fat, and 1.1 grams of sodium/1,000 kcal, respectively. Total HEI-05 ranges from 0 to 100 points and the higher the score, the more the diet complies with the 2005 DGA's, presuming a better diet quality [13]. For this analysis, missing values (*n* = 32) were assigned to participants with total daily energy (reported dietary intake) of either < 500 or > 5000 Kcal/day. There were missing values for physical activity and TV (*n* = 9) leaving *N* = 827 (224 AA, 246 HA, and 357 CA). There were *n* = 439 with and *n* = 388 without type 2 diabetes.

### 3.3. Statistical Analysis

Prior to analysis, all continuous variables were tested by the Kolmogorov-Smirnov test and transformed to achieve normality and linearity if needed. The general characteristics of the study population were compared by diabetes status and ethnicity using the Chi-squared test for categorical variables and the Student's *t*-test for continuous variables. To test the hypotheses, a binary variable for high versus normal hours per day of watching television was determined, using the 75th percentile for the combined group and allowing sufficient marginal means to compare by diabetes status. Ethnicity, diabetes status, dietary quality (HEI), and physical activity were the major independent variables for this study. Full logistic regression models included 2-way interactions for ethnicity, diabetes status, HEI and physical activity. Age, gender, obesity indicators (either waist circumference or BMI due to their collinearity), and smoking were included in the final model due to their clinical significance. A binary variable to indicate risk for diabetes was constructed with A1C = 6% as the cut-off. Potential confounders such as education and marital status were tested. Education was collapsed from 5 to 3 categories to include sufficient numbers in each category. The most parsimonious model was chosen by Hosmer and Lemeshow's recommendation for the elimination of adjustment variables (in this case education and marital status) with *P* values greater than 0.25 [20]. To assess differences in the dietary components of HEI across ethnicity, physical activity level, BMI, and TV viewing, ANOVA models with post hoc analyses were conducted. All analyses were performed by the *Statistical Package for the Social Sciences* (SPSS) version 19, (Chicago, IL, USA), and a *P*-value < 0.05 was considered significant.

## 4. Results

### 4.1. General Characteristics of the Study Population by Diabetes Status

Descriptive statistics of the study population are presented in Table 1. AA and HA with type 2 diabetes were significantly older than their counterparts without diabetes, whereas for CA there were no differences in age across diabetes status. As a whole, CA were the oldest, followed by HA, and AA. CA had more female participants; however, there were approximately equal numbers of males with and without diabetes within each ethnicity. Participants currently smoking did not differ by diabetes status; conversely, there were significant differences between ethnicities (analysis not shown). There were significant differences in education by ethnicity/race (*P* < 0.001) for the combined sample (with and without diabetes); AA had the highest percent with at least some college (55.1), followed by CA (36.8) and HA (32.9). CA had the highest percent with less than a high school diploma (49.6), followed by HA (46.3) and AA (16.0) (data not shown). Comparisons of each ethnicity by diabetes status are presented in Table 1. HA with diabetes had a lower education level than those without diabetes; however, AA and HA had no differences in education by diabetes status (AA and CA with type 2 diabetes had significantly higher BMI than those without diabetes. AA with type 2 diabetes) had significantly more participants who watched more than 4 hours of TV as compared to those without diabetes. CA with type 2 diabetes had higher HEI scores than those without diabetes. HA without diabetes were more physically active than those with type 2 diabetes. HA without diabetes had a significantly higher percent of persons at risk for diabetes (56.1) as compared to CA (41.2) and AA (37.5) as indicated by A1C ≥ 6.0 (*P*_ethnicity_ = 0.011) (analysis not shown).

### 4.2. Television Watching

The likelihood of viewing more than 4 hours of TV/day (“high TV”) is shown in Table 2. AA were more likely to watch more than 4 hrs of TV as compared to CA [OR = 5.30 (3.30, 8.49), *P* < 0.001 and HA [OR = 8.55 (4.88, 14.8), *P* < 0.001] (comparison of AA and HA was performed post-hoc by recoding ethnicity). Participants with diabetes were 1.62 (1.12, 2.34) times more likely (odds ratio) to watch more than 4 hours of TV as compared to those without diabetes (*P* = 0.010). A second model was conducted with A1C to account for differences in this risk factor for persons with and without diabetes. The results were consistent between the models for ethnicity. AA were still more likely to watch more than 4 hours of TV as compared to CA [OR = 5.13 (3.18, 8.25), *P* < 0.001]; however, adding A1C to the model negated differences by diabetes status [OR = 1.36 (0.85, 2.17), *P* = 0.199].

The outcome “high TV” was also explored to test the risk of high A1C by ethnicity stratified by diabetes status. Models, adjusted for age, gender, and BMI quartile, were significant [*χ* ^2^ (10) (*N* = 433) = 68.4, *P* < 0.001,* with diabetes* and *χ* ^2^ (10) (*N* = 386) = 33.9, *P* < 0.001, *without diabetes*]. AA were still more likely to watch more than 4 hours of TV as compared to CA [OR = 4.98 (2.70, 9.18), *P* < 0.001, *with diabetes* and OR = 3.56 (1.27, 9.94), *P* = 0.015, *without diabetes*]. Neither high A1C nor the interaction of high A1C with ethnicity was significant for those with or without diabetes [high A1C: *P* = 0.878, *with diabetes* and *P* = 0.503, *without diabetes*; high A1C by ethnicity: *P* = 0.644, *with diabetes* and *P* = 0.914, *without diabetes*] (data not shown).

### 4.3. Television Watching, HEI, and Physical Activity

The likelihood of diet score quartile and physical activity level with “high” TV viewing is presented in Table 3. Diet quality and physical activity were inversely related to “high TV viewing.” AA were 5.27 (3.09, 8.97) times more likely compared to CA and 8.06 (4.48, 14.5) times more likely (odds ratio) to watch more than 4 hours of TV as compared to HA (*P* < 0.001). The comparison of AA with HA was by post-hoc analysis, recoding ethnicity. 

### 4.4. Additional Analysis: Physical Activity and HEI

Diet quality was inversely associated with physical activity level as measured by the Chi-squared test and the *z*-test. The association of dietary quality with physical activity level was examined by the Chi-squared test [*χ* ^2^ (9) (*N* = 826) = 28.1, *P* = 0.001] and proportions were estimated by the *z*-test. The *z*-test results are shown in Table 4. 

### 4.5. Additional Analysis

Of interest were the differences in consumption of each food group of the HEI across ethnicities, physical activity, TV viewing, and obesity. First, the results of ANOVA and post-hoc analyses were performed for ethnicities. There were differences across ethnicities in dietary components of the HEI for all categories (*P* < 0.001). For most categories, post-hoc analysis showed differences between all three ethnicities from each other. The results are presented in Table 5. Healthier scores in SoFAAS and saturated fat were found for HA as compared to CA and AA. Of concern is the high sodium intake for CA. Proportionally, average servings from the fruit and vegetable groups were lower than those from the meat and bean group across ethnicities; however, HA had the highest total vegetable intake and the lowest meat and bean consumption as compared to CA and AA. 

Next, food groups were compared by ethnicity across PA quartiles by ANOVA. Significant differences were found in all fruit and vegetable groups (total fruit, *P* < 0.001; whole fruit, *P* < 0.001; total vegetables, *P* = 0.009; whole grains, *P* = 0.008; and, saturated fat, *P* = 0.030). Healthier eating patterns with increased PA were found only with the fruits and vegetables, and saturated fat categories, but not for whole grains. As PA increased, fruit and vegetable intake increased. For whole grains and saturated fat, healthier patterns were not observed with increased PA. The highest level of PA (Q4) as compared to the second level (Q2) had a lower consumption of whole grains (0.49, *P* = 0.008). The second level of PA (Q2) had a higher score (lower consumption) for saturated fat as compared to the first level (Q1) (0.75, *P* = 0.036). 

The third ANOVA and post-hoc analyses evaluated the HEI food groups across quartiles of TV viewing. There were differences in all food categories: total fruit, *P* < 0.001; total vegetables, *P* < 0.001; green vegetables, *P* < 0.001, grains *P* < 0.001, milk, *P* < 0.001, oils, *P* = 0.022; sodium, *P* = 0.005; and SoFAAS, *P* = 0.022, except the meat and bean group (*P* = 0.337) and the whole fruit group (*P* = 0.060). Those that watched TV, 3-4 hr/day (Q3) consumed less total fruits than those that watched the least TV (0–1.75 hr/day) (−0.46, *P* = 0.023). Total vegetable intake was lower for Q3 as compared to Q1 (−1.20, *P* < 0.001); Q4 as compared to Q3 (0.059; *P* = 0.003); and, Q4 as compared to Q1 (0.61, *P* = 0.003). Total grain intake was less in Q2 as compared to Q1 (−0.51, *P* < 0.001) and less for Q3 as compared to Q1 (−0.59, *P* < 0.001). Less whole grain consumption was associated with Q3 as compared to Q1 (−0.51, *P* = 0.003) and Q4 as compared to Q1 (−0.61, *P* = 0.001). More servings from the milk group were found for Q2 (0.92, *P* = 0.009) and Q3 (1.04,  *P* = 0.001) as compared to Q1. Lower intake of oils was associated with Q3 as compared to Q1 (−1.09, *P* = 0.016). A lower score for saturated fat (higher saturated fat consumption) was associated with Q3 and Q4 as compared to Q1 (−1.01, *P* = 0.001) and (−1.26, *P* < 0.001), respectively. For sodium, the highest quartile of TV viewing (Q4) had a lower score (higher than recommended intake) as compared Q1 (−0.79, *P* = 0.50), Q2 (−1.00, *P* = 0.007) and Q3 (−0.85, *P* = 0.021); although Q4–Q1 is marginally significant. The third quintile of TV viewing was associated with less adequate scores as compared to the lowest quintile (−1.63, *P* = 0.03) for SoFAAS. 

The food groups were also compared across quartiles of BMI. There were no significant differences in any food groups except for the meat and bean group (*P* < 0.001). The trend was as expected, the two highest quartiles of BMI consumed higher mean portions of meat and beans as compared to the two lower quartiles.

## 5. Discussion

Participants with and without diabetes who watched more than 4 hours of TV per day were more likely to have poorer quality diets and engage in less physical activity than those who viewed less hours of TV. Poorer quality diets, including higher amounts of processed foods (high calorie, high salt, low nutrition foods such as cold-cuts/lunch meat, sausages, fast food nuggets and burgers) and fried foods, refined grains, sugar, and lower fruit and vegetable intake, were associated with TV watching in adults [1, 2, 11]. The findings of a 6-year follow-up study suggest that participants free of diabetes who engage in prolonged TV viewing may be at risk for developing diabetes [2]. Our results were in agreement with several studies that found persons with type 2 diabetes have been shown not to follow recommended exercise and dietary practices independent of ethnicity. There were no significant differences in physical activity levels and dietary practices between a lower-income sample of approximately equal numbers of White non-Hispanics and AA with type 2 diabetes (*N* = 196) and 75% of both groups did not meet the government recommendations for physical activity and about 50% of both groups reported following recommended dietary practices (at that time was 30 minutes of exercise at least 3 times per week) [21]. These results were corroborated in a larger, multiethnic survey (*n* = 1560 Whites, *n* = 279 Blacks, and *n* = 125 Hispanics) of persons with diabetes enrolled in managed care where there were no differences across groups and approximately 55% reported exercising at least 3 times per week [22].

In our study AA with and without diabetes with poorest diet quality (lowest quartile of HEI-05) were most likely to watch more than 4 hours of TV as compared to those with better diet quality. Daily TV viewing has been estimated 7 hours for AA as compared to 5 hours for the total population [9]. Similar to the national trend, AA had a higher percent of participants classified in the obese categories. Obesity could be a limiting factor to performing physical activity due to physical and psycho-social factors. TV programs and commercials may influence eating behavior partly through a stimulus for food and overeating [23]. Certain populations may be more susceptible to the adverse effects of TV advertising [23]. More commercials for unhealthy foods were shown on TV programs aimed at AA audiences as compared to TV programs for the general population [24]. 

Our finding that physical activity was inversely associated with TV watching was in agreement with a large male population with type 2 diabetes (*N* = 37,918; aged 40–75 years) which indicated that men who spent more time watching TV were less likely to exercise [1]. Conversely our results were in discord with a population of approximately half Caucasian and half African American women (*N* = 189) [25] as well as a large study of French adults (*n* = 5028 men aged 45–60 and *n* = 7713 women aged 35–60) [26] and a large primarily Caucasian study of women (*N* = 50,277) [2]. 

Our results for no differences in physical activity comparing ethnicities may be inconclusive, since there has been a great variation in self-reported measures of physical activity and indices of cardiorespiratory fitness [27]; and references therein. The authors suggest that these variations may be greater for AA males as compared to other groups; however, they used a modified version of *CHAMPS Physical Activity Questionnaire among African-Americans* which assessed moderate and vigorous leisure activities, only [27]. In a US representative population of Hispanics, non-Hispanic Whites, and AA with diabetes, more AA males were exercising regularly as compared to other race and gender groups [28]. Yet, AA men had the lowest MET-minutes/week based on the *International Physical Activity Questionnaire* (IPAQ) suggesting discordance with perceptions and performance of exercise [28]. The IPAQ is a less-detailed physical activity questionnaire designed for developing countries and may not accurately capture physical activity for AA.

Our measure of physical activity included the total of all activities in the past year and the average per week was used for comparison. Accuracy of self-reported measurement of light activities, which occur during leisuretime and employment, may be a confounder in studies of physical activity level and TV viewing. Recreational activities and more intense sports are more accurately recalled than daily or lighter-intensity activities [27]. To its merit, the Modifiable Activity Questionnaire used in this study was designed to assess extreme levels of activity and validated to assess physical activity in populations with diabetes and at high risk for diabetes [6]. Since the majority of our participants engaged in light activity and did not meet the current recommendations for physical activity [29], a major strength of this questionnaire was the consideration of all types of physical activity performed by the individual including domestic choirs, transportation, employment, and leisure pursuits. 

### 5.1. Limitations

It is important to note the limitations of this research. The study was cross-sectional and TV viewing cannot be said to cause dietary and physical activity choices. Another possible limitation was nonresponse bias; participants who responded and agreed to be in this study may have different characteristics from those who were not reached or declined. Diet, physical activity and TV viewing were by self-report and the accuracy depends on the participant's ability to recall their behaviors. Consequently, there may have been over- or under-reporting biases. 

### 5.2. Recommendations for Interventions

Future studies are needed to establish prolonged TV watching in adult populations as a detrimental behavior and risk factor for the development of diabetes or diabetes-related complications. Although strategies to reduce TV watching have been proven effective among children, few trials have been conducted in adults [23]. A two-phase (3-week observation and 3-week intervention) randomized trial of 36 obese adults who normally watched a minimum of 3 hours of TV agreed to a 50% reduction in TV viewing per week using a lockout monitor showed promising results [30]. The intervention group showed an increase in energy expenditure without energy intake difference and decrease in BMI [30]. The findings from their study can be used to design future interventions. The investigators chose an intervention period of a short duration (3 weeks); albeit, increase in energy expenditure over time may prove to augment weight reduction. The influence of high-calorie, high-fat food advertisements may also be minimized by reducing TV viewing. Persons at risk for or having type 2 diabetes should be targeted for altering TV viewing habits in an effort to increase energy expenditure and reduce the influence of over-eating high-calorie, high-sodium, and high-fat-foods. In addition, strategies to substitute cheaper high calorie dense (low-nutrient) foods with cost-effective healthy foods are needed to reach communities in lower socio-economic neighborhoods.

## Figures and Tables

**Table 1:** General characteristics of the study population.

Variable	Ethnicity	Without T2D	With T2D	*P*-value
Age (years)	AA	51.1 ± 8.7	54.5 ± 10.2	0.007
Mean ± SD	HA	53.5 ± 10.7	57.8 ± 10.1	0.001
	CA	63.0 ± 10.9	65.2 ± 11.8	0.069

Gender *N* (%)	AA-F	54 (51.9)	64 (53.3)	0.893
	AA-M	50 (48.0)	56 (46.7)	
	HA-F	57 (50.0)	72 (54.5)	0.523
	HA-M	57 (50.0)	60 (45.4)	
	CA-F	113 (66.5)	116 (62.0)	0.439
	CA-M	57 (33.5)	71 (38.0)	

	AA	48 (40.0)	47 (36.4)	0.327
Currently smoking (yes) (*N*%)	HA	7 (5.8)	9 (6.5)	0.514
	CA	28 (16.2)	30 (16.0)	0.533

	AA	14 (13.5)	22 (18.2)	0.526
Education < HS degree/GED	HA	43 (37.7)	71 (53.8)	0.020
	CA	79 (46.2)	99 (52.7)	0.320

	AA	29 (27.9)	61 (51.3)	<0.001
BMI 4th Quartile *N* (%) (> 34 kg/m^2^)	HA	17 (15.0)	18 (13.6)	0.520
	CA	27 (15.9)	55 (29.4)	0.017

	AA	34 (32.7)	66 (55.0)	0.001
TV hours 4th quartile (> 4.0 hours) *N* (%)	HA	8 (7.0)	19 (14.4)	0.065
	CA	28 (16.5)	36 (19.3)	0.494

	AA	20 (19.2)	14 (11.7)	0.129
HEI 4th quartile (healthiest diet score) (> 59.3) *N* (%)	HA	38 (33.3)	37 (28.0)	0.782
	CA	34 (20.0)	63 (33.9)	0.004

	AA	35 (33.7)	29 (24.2)	0.404
PA (MET hr/wk) 4th quartile (highest activity) *N* (%)	HA	40 (35.1)	23 (17.4)	0.009
	CA	44 (25.9)	31 (16.6)	0.191

*Abbreviations*. AA: African American; HA: Haitian American; CA: Cuban American; T2D: type 2 diabetes; F: female; M: male; BMI: body mass index; HS: high school; GED: general equivalency diploma; TV: television; HEI: Healthy Eating Index; PA: physical activity; MET: metabolic equivalents.

*Notes.* Age was compared using the Student *t*-test and reported in mean ± SD. The Chi-squared test was used to for categorical data and reported as *N* (%) where % are within diabetes status. A *P* value of 0.05 was considered significant. Physical activity (PA) was measured in metabolic equivalents (MET) per week. MET = 1 kcal*·*kg^−1^ *·*h^−1^. One MET is the equivalent of sitting quietly.

**Table 2:** Likelihood of TV viewing more than 4 hour/day comparing ethnicities and diabetes status.

Independents	*B*	SE	df	*P*	OR	95% C.I. OR	
						Lower	Upper
Model 1							

Ethnicity	—	—	2	<0.001	—	—	—
AA	1.67	0.24	1	<0.001	5.29	3.30	8.49
HA	−0.37	0.26	1	0.160	0.69	−0.41	1.16
CA (reference group)	—	—	—	—	—	—	—
With diabetes	0.48	0.19	1	0.010	1.62	1.12	2.34

Model 2							

Ethnicity	—	—	2	<0.001	—	—	—
AA	1.63	0.24	1	<0.001	5.13	3.18	8.25
HA	−0.40	0.26	1	0.132	0.67	0.40	1.13
CA (reference group)	—	—	—	—	—	—	—
With diabetes	0.31	0.24	1	0.199	1.36	0.85	2.17

*Abbreviations*. AA: African Americans; *B*: coefficient; SE: standard error; df: degrees of freedom; Q: quartile; CA: Cuban American; HA: Haitian American.

*Notes.*  Television watching was reported to the nearest 0.25 hours. The dependent variable is ≥ 4.25 hours of TV versus ≤ 4.00 hours of TV. The 4th quartile of BMI is the reference.

*Model 1* was adjusted for age (*P* = 0.036), gender (*P* = 0.28), BMI quartiles (*P* = 0.331), currently smoking (*P* = 0.111), and education (*P* = 0.019). Participants who were older and had less education were more likely to watch more than 4 hours of TV per day as compared to their counterparts.

*Model 1 parameters*. *χ* ^2^ (11) = 112.8 (*N*  =  825), *P* < 0.001. Nagelkerke *R*-squared = 0.193 with 78.1% of the cases classified correctly.

*Model 2* included age (*P* = 0.051), gender (*P* = 0.228), BMI quartiles (*P* = 0.419), currently smoking (*P* = 0.082), education (*P* = 0.023), and an adjustment for high glycosylated hemoglobin: high A1C ≥ 6% (*P* = 0.321), diabetes status by high A1C (*P* = 0.134).

*Model 2 parameters*. *χ* ^2^ (13)  = 112.8 (*N* = 819), *P* < 0.001. Nagelkerke *R*-squared = 0.197 with 78.6% of the cases classified correctly.

**Table 3:** Effect of diet and physical activity on likelihood of viewing more than 4 hours/day of TV.

Independents	*B*	S.E	df	*P*	OR	95% C.I. OR	
						Lower	Upper
Quartiles HEI	—	—	3	0.014	—	—	—
Q1	0.80	0.29	1	0.006	2.22	1.25	3.92
Q2	0.25	0.29	1	0.337	1.29	0.74	2.25
Q3	0.69	0.28	1	0.015	1.98	1.14	3.45
Quartiles physical activity (MET/wk)	—	—	3	<0.001	—	—	—
Q1	1.18	0.28	1	<0.001	3.25	1.86	5.68
Q2	0.80	0.29	1	0.005	2.23	1.27	3.91
Q3	0.39	0.30	1	0.195	1.47	0.82	2.64
Ethnicity	—	—	2	<0.001	—	—	—
AA	1.66	0.27	1	<0.001	5.27	3.09	8.97
HA	−0.42	0.27	1	0.125	0.66	0.38	1.12
Diabetes (yes)	0.45	0.19	1	0.020	1.56	1.08	2.27

*Abbreviations*. BMI: body mass index; *B*: coefficient; SE: standard error; Q: quartile; HEI: Healthy Eating Index; AA: African American; HA: Haitian American.

*Notes*. Cuban Americans were the reference. The 4th quartile was the reference for HEI, physical activity, and BMI. Physical activity was measured in metabolic equivalents (MET) per week. MET = 1 kcal*·*kg^−1^ *·*h^−1^. One MET is the equivalent of sitting quietly. Television watching was reported to the nearest 0.25 hours. The dependent variable is ≥ 4.25 hours of TV versus ≤ 4.00 hours of TV. Education and currently married were tested. Education remained in the final model.

The model was adjusted for age (*P* = 0.058), gender (*P* = 0.705), BMI quartile (*P* = 0.207), currently smoking (*P* = 0.221), and education (*P* = 0.019). Compared to persons with at least college, those with less than a high school education were 1.54 times more likely to watch more than 4 hours of TV/day.

*Model parameters*. *χ* ^2^ (17) =  147 (*N* = 827), *P* < 0.001. Nagelkerke *R*-squared = 0.247 with 79.6% of the cases classified correctly.

**Table 4:** Cross tabulations of percentiles of HEI by percentiles of PA.

Quartiles of physical activity (MET/wk)		Quartiles of Healthy Eating Index, 2005				Total
		19.3–41.85	41.86–50.20	50.21–58.06	58.07–84.1
	*N*	67^a^	56^a,b^	50^a,b^	36^b^	209
1	% within PA	32.1	26.8	23.9	17.2	100.0
	% within HEI	32.5	27.1	24.2	17.5	25.3%

	*N*	46^a^	51^a^	46^a^	64^a^	207
2	% within PA	22.2	24.6	22.2	30.9	100.0
	% within HEI	22.3	24.6	22.2	31.1	25.1

	*N*	43^a^	43^a^	53^a,b^	69^b^	208
3	% within PA_	20.7	20.7	25.5	33.2	100.0
	% within HEI	20.9	20.8	25.6	33.5	25.2

	*N*	50^a^	57^a^	58^a^	37^a^	202
4	% within PA	24.8	28.2	28.7	18.3	100.0
	% within HEI	24.3	27.5	28.0	18.0	24.5

	*N*	206	207	207	206	826
Total	% within PA	24.9	25.1	25.1	24.9	100.0
	% within HEI	100.0	100.0	100.0	100.0	100.0

*Notes*. Each superscript letter denotes a subset of quartiles of Healthy Eating Index (HEI) categories whose column proportions do not differ significantly from each other at the alpha = 0.05 level. This option computes pairwise comparisons of column proportions and indicates which pairs of columns (for a given row) are significantly different, applying the Bonferroni correction. Different superscripts represent quartiles that differ at the alpha = 0.05 level. Quartiles of HEI score presented are rounded versions for informational purpose. Actual quartiles were calculated to the 0.001 place. Missing values (*n* = 32) were participants with total energies of either < 500 or > 5000 Kcal. The range of HEI-05 was 19.3–84.1 (based on a possible range of 0–100).

*Model parameters*. *χ* ^2^ (9) (*N* = 826) = 28.1, *P* = 0.001.

**Table 5:** Healthy Eating Index (HEI) food group scores by ethnicity.

Food group	Ethnicity	Mean (SD)	CA-HA	CA-AA	AA-HA
Total fruit	CA	2.68 (1.70)			
	HA	4.34 (1.25)	−1.57^∗^	−1.23^∗^	−0.34^∗^
	AA	3.91 (1.46)			

Whole fruit	CA	2.71 (1.94)			
	HA	4.20 (1.35)	−1.49^∗^	−0.93^∗^	−0.56^∗^
	AA	3.64 (1.63)			

Total vegetable	CA	1.59 (1.40)			
	HA	4.56 (0.89)	−2.97^∗^	−2.21^∗^	−0.76^∗^
	AA	3.64 (1.81)			

Green vegetables	CA	3.44 (1.54)			
	HA	3.54(1.60)	−0.94	1.25^∗^	−1.34^∗^
	AA	2.20 (1.50)			

Total grains	CA	2.58 (1.04)			
	HA	3.94 (1.18)	−1.35^∗^	−0.91^∗^	−0.44^∗^
	AA	3.49 (1.07)			

Whole grains	CA	1.23 (1.50)			
	HA	2.46 (1.59)	−1.23^∗^	−0.44^∗^	−0.79^∗^
	AA	1.66 (1.21)			

Milk	CA	5.51 (2.69)			
	HA	4.14 (3.03)	1.36^∗^	1.13^∗^	0.45
	AA	4.19 (2.72)			

Meat/beans	CA	5.17 (1.67)			
	HA	5.64 (1.39)	−0.47^∗^	−0.59^∗^	0.12
	AA	5.76 (1.29)			

Oils	CA	4.80 (1.48)			
	HA	5.64 (1.39)	−1.46	−1.90^∗^	0.44
	AA	5.76 (1.29)			

Saturated fat	CA	5.54 (2.80)			
	HA	8.26 (2.06)	−2.72^∗^	−0.83^∗^	−1.89^∗^
	AA	6.37 (2.51)			

Sodium	CA	7.85 (1.57)			
	HA	5.74 (3.11)	2.11^∗^	3.85^∗^	−1.73^∗^
	AA	4.00 (3.06)			

SoFAAS	CA	10.3 (4.69)			
	HA	13.4 (6.78)	−3.06^∗^	−0.20	−2.86^∗^
	AA	10.6 (6.85)			

*Ab* *br* *ev* *ia* *ti* *on* *s*. CA: Cuban American; HA: Haitian American; CA: Cuban American; SoFAAS: energy from solid fats, alcohol, and added sugars.

*Note*. An asterisk^∗^ denotes a significant difference at *P* < 0.05.
